# Creatine kinase is associated with reduced inflammation in a general population: The Tromsø study

**DOI:** 10.1371/journal.pone.0198133

**Published:** 2018-05-29

**Authors:** Svein Ivar Bekkelund, Stein Harald Johnsen

**Affiliations:** 1 Department of Neurology, University Hospital of North Norway, Tromsø, Norway; 2 Department of Clinical Medicine, The Arctic University of Norway, Tromsø, Norway; Rosalind Franklin University of Medicine and Science, UNITED STATES

## Abstract

**Background:**

Creatine kinase (CK) has been associated with reduced inflammation in obesity while inflammation is associated with obesity-related cardiovascular diseases. We investigated the relationship between CK and high sensitive C-reactive protein (hs-CRP) in a general population.

**Methods:**

CK and hs-CRP were measured in the population-based Tromsø study that included entire birth cohorts and random samples of citizens between 30–87 years of age. The analyses were performed sex-stratified in 5969 men and 6827 women.

**Results:**

CK correlated negatively with hs-CRP in men (r = -0.08, *P* <0.001) and women (r = -0.06, *P* <0.001). In univariable regression analyses, CK associated negatively with hs-CRP in men (*ß* = -0.14, 95% CI -0.19 to -0.10, *P* <0.001) and women (*ß* = -0.13, 95% CI -0.18 to -0.08, *P* <0.001). Mean CK declined from the 2. to the 4. quartiles of hs-CRP in both genders (*P* <0.001 for trends). There were positive correlations between CK and body mass index (BMI) in men (r = 0.10, *P* <0.001) and women (r = 0.07, *P* <0.001). Multiple regression analyses showed a 0.13 unit decrease in hs-CRP (mg/dl) per unit CK increase in men (95% CI -0.35 to -0.20) and 0.29 mg/dl in women (95% CI -0.36 to -0.21) when adjusted for age, BMI, lipids, s-glucose, s-creatinine, transaminases and coronary heart disease.

**Conclusion:**

CK were inversely and independently associated with hs-CRP in a general population. These data provide evidence that CK might have anti-inflammatory properties, but the mechanism and clinical implications are unclarified.

## Introduction

Creatine kinase (CK), an enzyme promoting cellular energy metabolism mainly in skeletal muscles, has been associated with inflammation. Mechanisms to explain this are not established, but obesity is a possible link [1]. Plasma levels of inflammatory biomarkers predict obesity [2–4], and obesity-related inflammation is associated with increased risk of cardiovascular diseases (CVD) and CVD-mortality [5]. Pro-inflammatory metabolites (adipokines) produced by adipocytes seem to be an important mechanism [6], but there is additional evidence for a contribution of skeletal muscle in the inflammatory process related to obesity [7]. As opposed to adipocytes, release of biologically active metabolites (myokines) from myocytes has been related to reduced obesity-related inflammation [8]. There is therefore a possible interaction or “crosstalk” between myocytes and adipocytes involved in the process of adiposity [9, 10].

CK is associated with body mass index (BMI) in population-based studies [11, 12] and in a case-control study primarily designed to investigate the relationship between CK and blood pressure [13]. Inflammatory markers in obese women with reduced muscle mass and increased fat mass (sarcopenic obesity) were associated with higher CK [14]. Inflammation representing a shared mechanism for release of CK to the circulation may be attributed to physical activity. Accordingly, a higher CK-response was observed after performing eccentric exercise in elderly obese subjects with increased level of interleukin 6 [15]. Furthermore, CK along with lean mass were inversely and independently associated with C-reactive protein (CRP) in a sample of 454 overweight and obese individuals, supporting an anti-inflammatory role for CK [1]. Studies about how CK activity at rest associates with inflammation should also include non-obese populations. We therefore hypothesized an independent relationship between CK and CRP in the general population.

## Material and methods

### Study participants

This project is based on data from the 6^th^ Tromsø Study, a prospective population-based study that started in 1974. Originally, the Tromsø Study focused on CVD risk factors, but a number of variables linked to a variety of issues have been included since then [16]. Participants in a previous survey (4^th^ Tromsø study), a 10% random sample from age groups 30–39, all participants aged 40–42 and 60–87. The data were collected from October 2007 to 19 September 2008. Totally, 12984 men and women participated. The population was mainly Causation (87.3% ethnic Norwegians, 1.6% of Sami ethnicity, 1.3% of Finnish origin, 2.2% of other ethnicities, and 7.6% without information about ethnicity) [16]. Written consent was obtained from all participants, and the Norwegian Committee for Medical and Health Research Ethics (REC) approved the study.

### Measurements

Hs-CRP was analysed in thawed aliquots after storage at − 20 °C with a particle-enhanced immunoturbidimetric assay on a Modular P (Roche Hitachi, Mannheim, Germany), with reagents from Roche Diagnostics (Mannheim, Germany). Samples were analysed in batches during the time of the survey. The lower detection limit of the high-sensitivity CRP assay was 0.03 mg /L and measurements of CRP lower than 0.03 mg /L were set at this value. The analytical coefficient of variation for CRP levels between 0.1 mg /L and 20 mg /L was < 4%.

Serum-CK was analysed consecutively within 6 hours after the phlebotomies in an automated clinical chemistry analyzer (Modular P, Roche) by photometry, using an enzymatic method (CK-NAC, Roche Diagnostics, Mannheim, Germany). The analytic variation coefficient was ≤ 1.6%. The standard cut-off limits for CK developed by the Nordic Reference Interval Project (NORIP) were: Men between 18–50 years (50–400 U/L); Men ≥ 50 years (40–280 U/L); Women (35–210 U/L) [17]. Men (N = 7) and women (N = 16) with CK ≥ 1000 U/L were regarded as outliers and therefore excluded. Non-fasting S-glucose was obtained. Determination of glycosylated hemoglobin (HbA1C) in EDTA whole blood was based on an immunoturbidometric assay (UNIMATES, F. Hoffmann-La Roche AG). The HbA1c percent value was calculated from the HbA1C/Hb ratio. Serum total cholesterol was analysed by an enzymatic colorimetric method using a commercially available kit (CHOD-PAP, Boehringer-Mannheim, Mannheim, Germany). Serum high-density lipoprotein (HDL) cholesterol was measured after precipitation of lower-density lipoproteins with heparin and manganese chloride. All the analyses were done at the Department of Clinical Biochemistry, University Hospital of North Norway. According to the standard procedure in the Tromsø study, height and weight were measured wearing light clothing without shoes to the nearest 0.1 cm and 0.1 kg using an automatic device, and BMI calculated as weight (kg) divided by height squared (m^2^). Waist- and hip circumference were measured and waist-to-hip ratio calculated. Information on diabetes, use of lipid-lowering drugs and coronary heart disease was obtained from standard questionnaires in the Tromsø study. Coronary heart disease was registered as a case when participants reported previous heart attack. Hypertension was defined as systolic blood pressure ≥ 140 mmHg, diastolic blood pressure ≥ 90 mmHg or ever use of antihypertensive drugs. Diabetes was defined as HbA1c ≥ 6.5% or use of antidiabetic drugs.

### Statistical analysis

We performed the statistical analysis using SPSS software version 23 (SPSS INC, Chicago, Illinois, USA). Endpoint variables were visually inspected by histograms, and kurtosis and skewness calculated to ascertain normal distribution. The histograms showed right-sided skewness in both variables for men and women. Serum CK (men: skewness 2.7, kurtosis 11.9; women: skewness 3.7, kurtosis 25.4) and hs-CRP (men: skewness 7.5, kurtosis 82.8; women: skewness 9.5, kurtosis 167.0) confirmed non-Gaussian distribution of the data. Subsequent analyses of log CK (men: skewness 0.4, kurtosis 0.5; women: skewness 0.5, kurtosis 1.2) and log hs-CRP (men: skewness 0.7, kurtosis 0.7; women: skewness 0.5, kurtosis 0.2) and inspection of histograms showed normal distribution of the variables. We therefore used log-transformed data for CK and hs-CRP in the analyses.

Descriptive data are presented as mean ± standard deviations (SD) or numbers and frequencies in men and women separately. Two-sided Student´s t-test was used to calculate differences between means and χ^2^- test to compare frequencies of data within groups (dichotomous data). Due to different cut-off levels of CK, its association to hs-CRP were analysed separately for men and women. To assess linear trends of CK across quartiles of hs-CRP, we used ANOVA. By multiple regression analysis, possible confounders were tested and adjusted for with hs-CRP as the dependent variable and CK, age, BMI, HDL- and LDL-cholesterol [18], s-glucose [19], creatinine, transaminases and coronary heart disease [20] as independent variables. The independent variables were included in the multivariable model if reported in the literature as associated with CRP or appeared significantly related to CRP in univariable regression analyses in the present samples. Repeated tests replacing BMI with alternative obesity-parameters (waist circumference and hip-to-waist ratio) were performed to further elucidate associations with obesity. The level of significance was set at 5%.

## Results

In this population, men and women were of the same age, but men had higher scores on obesity-parameters than women (Table 1). About 20% of both sexes were obese (Table 1). Log CK and log hs-CRP were higher in men, and men more commonly reported coronary heart disease (heart attack), diabetes, hypertension and use of lipid-lowering drugs than women did (Table 1). Mean CK (SD) in male participants currently using lipid-lowering drugs was 2.09 (0.22) U/L (n = 992) compared to 2.10 (0.23) U/L in the others (n = 4977, *P* = 0.17). The corresponding values for women were 1.94 (0.20) U/L (*n* = 845) vs. 1.94 (0.21) U/L in others (n = 5982, *P* = 0.62). There were positive correlations between CK and BMI in (r = 0.10, *P* <0.0001) and women (r = 0.07, *P* <0.0001).

**Table 1:** Clinical characteristics of the subjects presented as numbers (%) or mean (SD).

Variables	Men (n = 5969)	Women (n = 6827)	*P*
Age (years)	57.4 (12.3)	57.4 (13.0)	1.0
Height (cm)	176.9 (6.9)	163.3 (6.5)	<0.0001
Weight (kg)	85.4 (13.3)	70.9 (13.0)	<0.0001
BMI (kg/m^2^)	27.3 (3.8)	26.6 (4.7)	<0.0001
Waist circumference (cm)	99.5 (10.6)	90.9 (12.2)	<0.0001
Hip circumference (cm)	104.4 (6.6)	103.9 (9.1)	0.001
Waist-to-hip ratio	0.95 (0.07)	0.87 (0.09)	<0.0001
Obesity (BMI ≥30 kg/m^2^)	1234 (20.7)	1397 (20.5)	0.69
Diabetes mellitus, n (%)	327 (5.5)	306 (4.5)	0.01
Use of lipid lowering drugs	992 (16.6)	845 (12.4)	<0.0001
Hypertension	2083 (34.6)	1949 (28.6)	<0.0001
Coronary heart disease	482 (8.1)	199 (2.9)	<0.0001
S-creatinine (μmol/l)	78.7 (15.9)	62.7 (12.5)	<0.0001
S-glucose (mmol/l)	5.38 (1.38)	5.12 (1.05)	<0.0001
S-HbA1C (%)	5.70 (0.71)	5.60 (0.60)	<0.0001
S-total cholesterol (mmol/l)	5.50 (1.06)	5.70 (1.11)	<0.0001
S-HDL-cholesterol (mmol/l)	1.35 (0.38)	1.65 (0.44)	<0.0001
S-LDL-cholesterol (mmol/l)	3.56 (0.93)	3.55 (0.97)	0.63
S-CK (U/L)	147.8 (96.2) (IQR12.0–981.0)	98.6 (60.1) (IQR10.0–871.0)	<0.0001
Log CK (U/L)	2.10 (0.23)	1.94 (0.20)	<0.0001
High CK ^a^	319 (5.3)	276 (4.0)	<0.0001
Hs-CRP (mg/dl)	2.5 (4.7) (IQR 0.1–82.9)	2.5 (4.6) (IQR 0.1–136.6)	0.66
Log hs-CRP (mg/dl)	0.15 (0.41)	0.14 (0.44)	0.01
S-ALT (U/L)	32.6 (19.4)	26.3 (25.6)	<0.0001
S-AST (U/L)	27.9 (10.0)	25.3 (14.2)	<0.0001

CK, creatine kinase; hs-CRP, high sensitive C-reactive protein; BMI, body mass index; HDL, high-density lipoprotein; LDL; low-density lipoprotein; ALT, alanine transaminase; AST, aspartate transaminase

^a^ Reference limits for normal CK: Men < 50 years: 50–400 U/L, men ≥ 50 years: 40–280 U/L, women: 35–210 U/L

**Fig 1:**
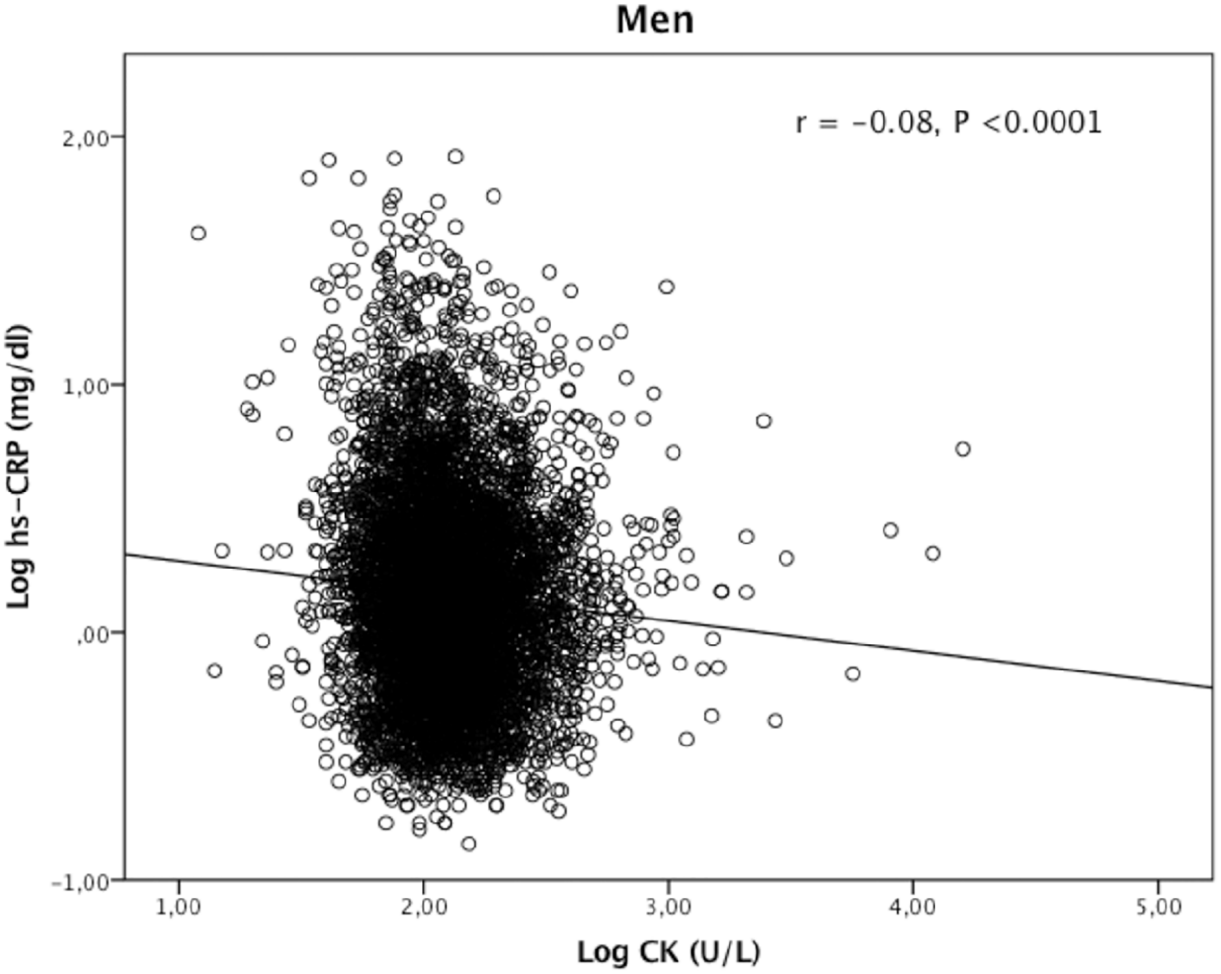
Correlation between creatine kinase (CK) and high sensitive C-reactive protein (hs-CRP) in 5969 men from a general population.

**Fig 2:**
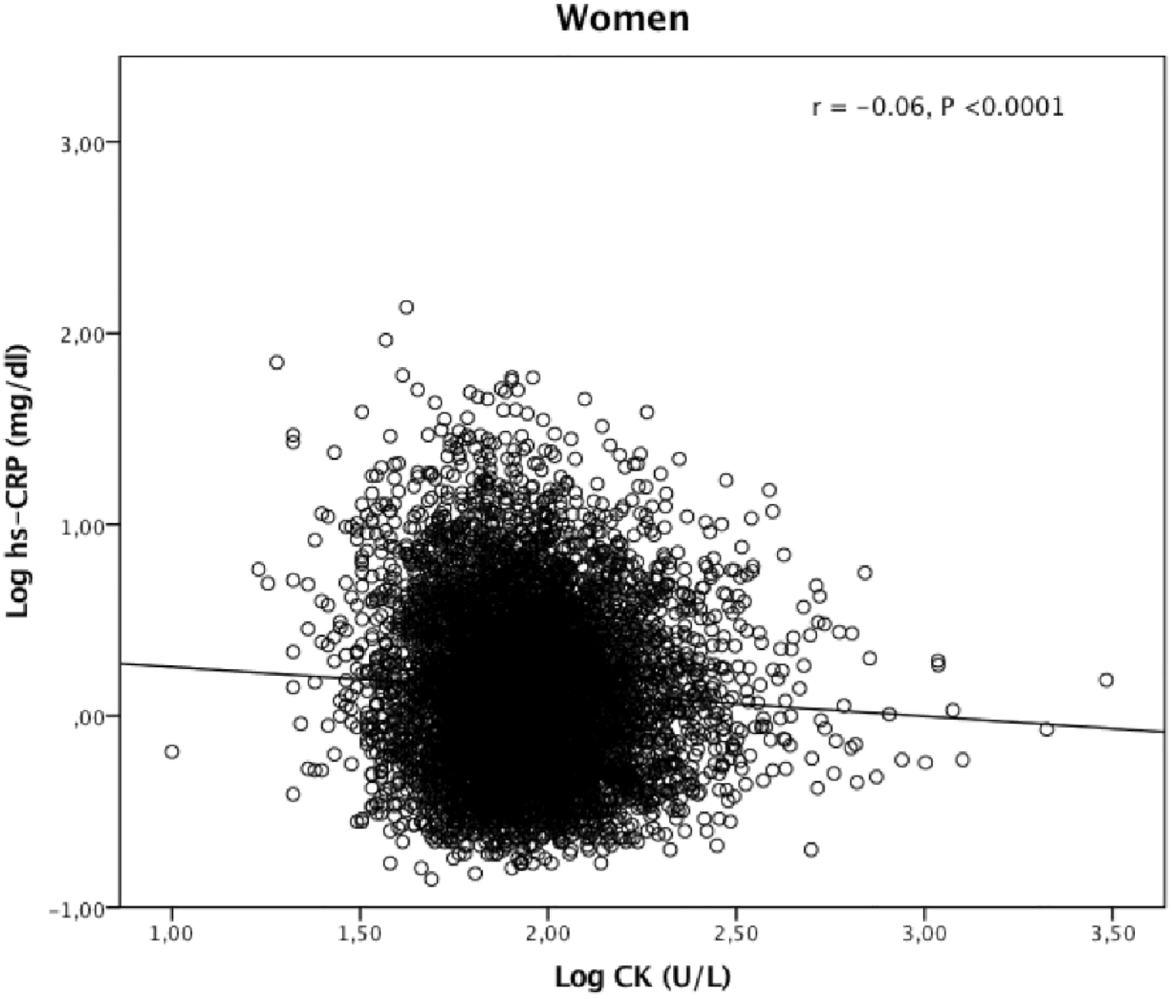
Correlation between creatine kinase (CK) and high sensitive C-reactive protein (hs-CRP) in 6827 women from a general population.

CK correlated negatively with hs-CRP in both genders (Figs 1 and 2). Univariable linear regression analyses showed CK to be negatively associated with hs-CRP in men and women (*P* <0.0001) (Table 2). Conversely, obesity-parameters, s-glucose and HbA1C were positively associated with hs-CRP. S-total cholesterol and LDL cholesterol were positively associated with hs-CRP in women only, while HDL-cholesterol associated negatively with hs-CRP in both genders (Table 2). Mean hs-CRP (SD) in men with coronary heart disease was 0.20 (0.43) mg/dl compared to 0.15 (0.41) mg/dl in the others (*P* = 0.006). The female data were 0.21 (0.41) mg/dl respectively 0.13 (0.44) mg/dl (*P* = 0.005). Diabetes mellitus and creatinine were not associated with hs-CRP or CK (data not shown).

**Table 2:** Associations between hs-CRP, CK and potential confounders.

	Log hs-CRP in men (mg/dl)		Log hs-CRP in women (mg/dl)	
	ß ^a^ (95% CI)	*P*	ß ^a^ (95% CI)	*P*
Log CK (U/L)	-0.14 (-0.19 to -0.10)	<0.0001	-0.13 (-0.18 to -0.08)	<0.0001
Age (years)	0.005 (0.005 to 0.006)	<0.0001	0.007 (0.006–0.007)	<0.0001
BMI (kg/m^2^)	0.029 (0.026 to 0.031)	<0.0001	0.040 (0.038 to 0.042)	<0.0001
Waist circumference (cm)	0.011 (0.010 to 0.012)	<0.0001	0.015 (0.014 to 0.016)	<0.0001
Waist-to-hip ratio	0.011 (0.010 to 0.012)	<0.0001	1.93 (1.79 to 2.08)	<0.0001
S-gluccose (mmol/l)	0.022 (0.015 to 0.030)	<0.0001	0.058 (0.049 to 0.068)	<0.0001
S-HbA1C (%)	0.099 (0.084 to 0.114)	<0.0001	0.173 (0.156 to 0.190)	<0.0001
S-total-cholesterol (mmol/l)	0.004 (-0.006 to 0.014)	0.43	0.036 (0.027 to 0.045)	<0.0001
S-HDL-cholesterol (mmol/l)	-0.193 (-0.220 to -0.165)	<0.0001	-0.231 (-0.255 to -0.208)	<0.0001
S-LDL-cholesterol (mmol/l)	0.010 (-0.001 to 0.022)	0.071	0.055 (0.044 to 0.066)	<0.0001
S-creatinine (μmol/l)	0.002 (0.001–0.002)	<0.0001	0.002 (0.001–0.003)	<0.0001
S-ALT (U/L)	0.002 (0.001–0.003)	<0.0001	0.002 (0.001–0.002)	<0.0001
S-AST (U/L)	0.003 (0.002–0.005)	<0.0001	0.003 (0.002–0.004)	<0.0001

CK, creatine kinase; hs-CRP, high sensitive C-reactive protein; BMI, body mass index; HDL, high-density lipoprotein; LDL; low-density lipoprotein; ALT, alanine transaminase; AST, aspartate transaminase

^a^ The regression coefficient (*ß*) and 95% CI expressed in mg/dl for a 1-unit increase in continuous variables

CK declined significantly from quartiles 2 to 4 of hs-CRP in both sexes (Table 3) and CK was inversely and independently associated with hs-CRP in men and women when adjusted for age, BMI, glucose, creatinine, transaminases, lipids and coronary heart disease (Table 4). In men, a 1-unit increase in CK was associated with 0.13 mg/dl lower hs-CRP (95% CI -0.35 to -0.20). In women, the corresponding values were 0.29 mg/dl (95% CI -0.36 to -0.21). A multivariable-adjusted sensitivity analysis excluding participants with incidents of coronary heart disease (men: n = 482; women: n = 199) did not change the results (data not shown). Neither did inclusion of LDL-cholesterol (replacing HDL-cholesterol) or waist circumference and waist-to-hip ratio (replacing BMI) change the results (data not shown). Mean (SD) hs-CRP in the male subgroup with self-reported coronary heart disease (n = 482) was 0.20 mg/dl (0.43) compared to 0.15 mg/dl (0.41) in the others (n = 5487, *P* = 0.006). The equivalent data for women (n = 199) were 0.21 mg/dl (0.41) vs. 0.13 mg/dl (0.44), (n = 6628, *P* = 0.008). CK correlated with creatinine in men (*r* = 0.047, *P* < 0.001) and women (*r* = 0.113, *P* < 0.001). Additionally, CK correlated inversely with glucose in men (*r* = -0.037, *P* < 0.001) but not in women (*r* = 0.009, *P* = 0.48).

**Table 3:** CK in quartiles of hs-CRP.

Log hs-CRP quartiles (mg/dl)					
	Q 1	Q 2	Q 3	Q 4	*P* for trend
N (men)	1502	1484	1464	1519	
Log hs-CRP (mg/dl)	≤-0.14	-0.13–0.10	0.11–0.38	≥0.39	
Log CK (U/L)	2.11 (0.21)	2.11 (0.23)	2.10 (0.23)	2.08 (0.22)	<0.0001 ^a^
N (women)	1687	1717	1718	1705	
Log hs-CRP (mg/dl)	≤-0.20	-0.19–0.09	0.10–0.41	≥0.42	
Log CK (U/L)	1.95 (0.20)	1.95 (0.21)	1.94 (0.21)	1.92 (0.22)	<0.0001 ^a^

CK, creatine kinase; hs-CRP, high sensitive C-reactive protein, Q1, first quartile; Q2, second quartile; Q3, third quartile; Q4, fourth quartile, CI; confidence interval

^a^ The trends analysed by ANOVA were significant from quartiles 2 to 4

**Table 4:** Associations between hs-CRP (dependent variable) and independent variables in men and women.

	Log hs-CRP in men (mg/dl)		Log hs-CRP in women (mg/dl)	
	ß (95% CI)	*P*	ß (95% CI)	*P*
Log CK (U/L)	-0.13 (-0.35 to -0.20)	<0.0001	-0.29 (-0.36 to -0.21)	<0.0001
Age	0.005 (0.004 to 0.007)	<0.0001	0.004 (0.002 to 0.005)	<0.0001
BMI	0.027 (0.022 to 0.032)	<0.0001	0.037 (0.034 to 0.041	<0.0001
S-HDL-cholesterol (mmol/l)	-0.14 (-0.18 to -0.09)	<0.0001		
S-LDL-cholesterol (mmol/l)			0.008 (-0.008 to -0.024)	0.34
S-glucose (mmol/l)	0.001 (-0.011 to 0.013)	0.86	0.019 (0.005 to 0.034)	0.01
Coronary heart disease	-0.09 (-0.15 to -0.04)	0.001	-0.07 (-0.16 to -0.02)	0.14
S-creatinine (μmol/l)	0.001 (0.000 to 0.002)	0.007	0.001 (-0.001 to 0.002)	0.42
S-ALT (U/L)	-0.001 (-0.002 to -0.005)	0.34	-0.001 (-0.003 to -0.000)	0.054
S-AST (U/L)	0.005 (0.002 to 0.007)	<0.0001	0.004 (0.001 to 0.006)	0.003
*R*^*2*^ = 0.13		*R*^*2*^ = 0.19		

CK, creatine kinase; hs-CRP, high sensitive C-reactive protein; BMI, body mass index; HDL, high-density lipoprotein; LDL; low-density lipoprotein; CI; confidence interval; ALT, alanine transaminase; AST, aspartate transaminase

## Discussion

This study demonstrated an inverse and significant association between CK and hs-CRP in the general population. After adjusting for obesity-related variables, s-glucose, HbA1C, creatinine, transaminases and lipids, CK remained independently associated with hs-CRP. CK may hypothetically inhibit inflammation in humans, but the mechanism and clinical implications are uncertain.

This result parallels the finding in a previous clinical study comparing CK and CRP in obese individuals [1]. In that study, which motivated the present one, CK and lean body mass were negatively associated with CRP indicating an inhibitory effect on obesity-related inflammation by CK and/or by other muscular metabolites [1]. The present study therefore supports an inverse relationship between CK and CRP by its presence in the general population, but an influence of obesity is not confirmed from these data. Nevertheless, BMI was associated with elevated CK as proven by others [11], and CRP was positively associated with BMI. In a recent report, CK was inversely associated with fat mass in obese subjects indicating a favourable role for CK in the process of adiposity [21]. In subsequent studies, body composition examinations comparing body fat and lean mass with CRP should be performed in general populations to further elucidate the mechanism(s). BMI may fail to predict obesity because it does not distinguish between fat and muscle content [22, 23]. Nor does it reflect body fat distribution. Likewise, those with increased adipose tissue and reduced muscle mass (sarcopenic phenotype) are at risk of adverse outcome, but may be overlooked when using BMI as an obesity marker [24].

The cascade of events leading to atherosclerosis and CVD is complex, but macrophage influx in adipose tissue with local inflammation [25], release of adipokines [26] and liver-induced CRP are important reactions. In the present study, hs-CRP was positively associated with BMI confirming findings from prior reports [27, 28]. Hs-CRP was also higher in participants with coronary heart disease. In studies with examination of body composition, inflammation has been positively associated with fat mass and negatively associated with lean mass [7]. In a longitudinal cohort study, high CRP and interleukin (IL)-6 were associated with loss of lean mass [29]. Studies on the relationship between CK and adipokines have been reported in relation to muscle exercise [15], but not at rest in obesity or in the general populations. Thus, adipocyte/myocyte activity may be integrated. Myokines such as myostatin, IL-4, -6, -7, -15 are probably involved in the local energy metabolism in brown fat [30, 31]. Impact of lean mass on obesity in younger age groups without sarcopenic obesity is less investigated. Furthermore, skeletal muscle is an important site of insulin action, and increased lean mass is associated with better insulin sensitivity and reduced inflammation [32]. CK plays an important role in the cellular energy metabolism in skeletal muscle, and additionally, creatine enhances respiration rate in brown adipocyte mitochondrion, and thereby increases cellular heat and energy expenditure [33]. This indicates a possible mechanism for how CK might interfere with obesity, and why it is worth hypothesizing an association between CK and inflammation. CK has not been measured in fat tissue, however.

High CK may have many causes [34] and it is important to control for contributing effects of accompanying disorders. Earlier studies focusing on conditions associated with hyperCKemia and inflammation show that high CK may possibly be explained by inflammatory myopathies in some cases [35]. Such diseases are rare in general populations however, and they seldom go unrecognized because of typical symptoms. Additionally, CK showed lower values in patients with inflammatory rheumatic diseases [36, 37]. Inflammatory activity, muscle mass and steroid use were factors associated with reduced CK in one study [37]. These studies indicate that inflammation may initiate a process leading to reduction in CK levels but the cause-effect direction in the CK-CRP connection is still unknown, especially when it comes to the relationship between CK and obesity. By excluding participants with CK ≥ 1000 U/L in this study, we reduced the possibility of including patients with diseases that affect CK, such as neuromuscular disorders and inflammatory conditions, but use of steroids or other compounds with a potential to influence CK and CRP levels is unknown except for lipid-lowering drugs. This population should therefore be well designed to study population aspects of CK activity at rest, but lack of information about muscular strain prior to blood withdrawal in the participants is nevertheless a limitation to the study. Hypothetically, inflammation may facilitate release of CK to the circulation, especially during muscular exertion [15]. Whether CK exerts any direct role on reducing hs-CRP need experimental evidences.

## Conclusion

CK and CRP were inversely related in men and women in the prospective population-based Tromsø study. This supports the hypothesis that CK may have an anti-inflammatory effect, but the direction of the relationship and clinical implications are yet to be investigated. CK measurement should be considered in cases with inflammation of unknown origin.
